# Effects of Orientations, Aspect Ratios, Pavement Materials and Vegetation Elements on Thermal Stress inside Typical Urban Canyons

**DOI:** 10.3390/ijerph16193574

**Published:** 2019-09-24

**Authors:** Gabriele Lobaccaro, Juan Angel Acero, Gerardo Sanchez Martinez, Ales Padro, Txomin Laburu, German Fernandez

**Affiliations:** 1 Department of Architecture and Technology, Norwegian University of Science and Technology, 7491 Trondheim, Norway; 2 TECNALIA, Energy and Environmental Division, Parque Tecnologico de Bizkaia, Edificio 700, 48160 Derio, Bizkaia, Spain; juanangel@smart.mit.edu (J.A.A.); ales.padro@tecnalia.com (A.P.); txomin.laburu@tecnalia.com (T.L.); jgerman.fernandez@tecnalia.com (G.F.); 3 CENSAM, Singapore-MIT Aliance for Reasearch and Tecnology (SMART), 1 Create Way, Singapore #09-03, Singapore; 4 Department of Technology, Management and Economics, Technical University of Denmark, Marmorvej 51, Building FN Byen, 2100 Copenhagen, Denmark; gsama@dtu.dk

**Keywords:** outdoor thermal comfort, PET, ENVI-met, urban canyon, coastal, mid-latitude regions

## Abstract

The analysis of local climate conditions to test artificial urban boundaries and related climate hazards through modelling tools should become a common practice to inform public authorities about the benefits of planning alternatives. Different finishing materials and sheltering objects within urban canyons (UCs) can be tested, predicted and compared through quantitative and qualitative understanding of the relationships between the microclimatic environment and subjective thermal assessment. This process can work as support planning instrument in the early design phases as has been done in this study that aims to analyze the thermal stress within typical UCs of Bilbao (Spain) in summertime through the evaluation of Physiologically Equivalent Temperature using *ENVI-met*. The UCs are characterized by different orientations, height-to-width aspect ratios, pavement materials, trees’ dimensions and planting pattern. Firstly, the current situation was analyzed; secondly, the effects of asphalt and red brick stones as streets’ pavement materials were compared; thirdly, the benefits of vegetation elements were tested. The analysis demonstrated that orientation and aspect ratio strongly affect the magnitude and duration of the thermal peaks at pedestrian level; while the vegetation elements improve the thermal comfort up to two thermophysiological assessment classes. The outcomes of this study, were transferred and visualized into green planning recommendations for new and consolidated urban areas in Bilbao.

## 1. Introduction

The current world population is expected to reach 8.5 billion by 2030, 9.7 billion in 2050 and 11.2 billion in 2100 [1]. This rapid growth of the world’s population means that in the near future, more than half of all people will live in cities and this trend will inevitably have a strong impact on the sustainability and the energy costs of the built environment [2]. As a consequence of the global trend towards rapid and uncontrolled urbanization an increase in the magnitude of urban heat island (UHI) phenomena can be expected, together with an alteration of local patterns [3,4]. The natural and artificial morphology have influence also on the meteorological parameters such as air temperature (T_a_), relative humidity (RH), wind velocity (W_s_), mean radiant temperature (T_mrt_), surface temperature (T_s_), long– and short-wave (Sw) radiation which affect the thermal comfort of people living in cities [5,6]. Thermal comfort represents the conditions of mind that expresses satisfaction with the thermal environment and is assessed by subjective evaluation [7] by using thermal indices that associate microclimatic conditions with human thermal sensations derived from the energy balance of the human body. In the literature extended dedicated reviews [8,9,10] present and compare [11,12,13] the existing thermal comfort indices, which are used in many bioclimatology, applied climatology and city case studies applications [6,14,15,16,17,18,19]. The Physiologically Equivalent Temperature (PET) [6], the Standard Effective Temperature [7], and the predicted mean vote (PMV) [20] are some of the most commonly used indexes for outdoor thermal comfort [21]. However, the use of the Universal Thermal Comfort Index (UTCI) [11] is increasing in current studies. Since thermal perception/stress depend on psychological factors and cultural characteristics that affect acclimatization of an individual to a certain climate [2], thermal comfort indexes are usually modified and calibrated across different climatic regions and cultures [22,23]. Furthermore, it is important to consider that the relationship between high temperatures and urban settings have severe health impacts [24,25], including mortality, as has occurred recently in temperate regions [26,27]. In 2003, Europe experienced a devastating long summer heat wave that affected most of the continent and caused up to 15,000 deaths only during the first week of August and an estimated total mortality of around 70,000 [28,29,30]. Merte [24] estimated heat-related deaths in Europe from 1960 to 2014 at around 28,000 (on average) annually. Furthermore, the combination of global warming, aging and continuing urbanization is likely to render urban inhabitants increasingly vulnerable to extreme weather conditions in the absence of adequate adaptation strategies [31,32,33]. According to climate projections, a consistent increase in the number of heat waves events [34], their frequency [35] and intensity [36,37] is expected. In this regard, the role of prevention (e.g. in the form of comprehensive Heat-Health Action Plans) and of municipalities as key implementers of it has become crucial, as local governments are increasingly adopting long-term mitigation and adaptation interventions to face the impacts of such climate-related extremes. Therefore, it is also important to consider heat exposure indicators for health impact assessment, which have mostly been studied at either the population level in observational studies or in occupational settings. Regarding heat risk perception, these seem to be one of the critical areas hindering the effectiveness of public health protection against heatwaves [38]. These behavioral factors working against health protection from heat seem to be, however, highly context specific [39], and in this sense urban greening may not by itself be assumed to universally protect against high temperatures. Rather, it depends on how much actual temperatures may be reduced both outdoors and indoors (where people tend to spend most of their time), and on how people interact or not with the green spaces, which is in turn related with a wide range of factors from accessibility to socioeconomic status, as well as several behavioral and psychological mediators [40,41,42]. Greening as urban mitigation strategy in highly dense built environments is being more and more used to improve the quality of urban spaces and to benefit the local climate conditions [43,44] and humans’ thermal comfort [45,46,47]. The variations of sun and shade spaces, and changes in W_s_, T_a_, RH and other climate parameters inevitably affect the local climate characteristics of urban environment as well as the citizens’ thermal stress, given their direct exposure to these factors. Therefore, the role of architects, urban planners, landscapers, politicians, developers and engineering firms is very important given that political and design decisions can consistently improve the quality of urban microclimate [48] and of the livability of urban public spaces [2,49,50]. In this framework, taking into account the outdoor human thermal comfort dimension already in the early design phases, can lead to a more holistic view of sustainable urban development [51,52,53,54,55] and health impacts [56,57,58,59]. In this scenario, the use of numerical models can help to simulate local conditions and predict the effect of design and planning interventions. In that sense, the multitude of different finishing materials and sheltering objects produce a very distinct pattern of climate conditions, especially within street canyons [60]. This happens mostly during the daytime, in which the combination of high temperatures and intense solar radiation create high heat stress conditions [61]. Therefore, it is becoming a common practice to inform technical (i.e., urban planners, landscape designers, architects, engineers etc.) and non-technical actors (i.e., urban decision-makers, legislators, stakeholders, citizens etc.) about the effectiveness of new or refurbishment urban interventions by using modelling tools. Their use enables the benefits of various design and planning alternatives to be quantitatively and qualitatively tested, predicted and compared, on different perspectives from thermal comfort to economic and legislative field. This process works ideally as support instrument during the early design phases where the most relevant and critical design decisions are taken. In this study microclimate analyses were conducted in typical urban street canyons (UCs) of Bilbao (Spain) to predict the benefits provided by vegetation elements (e.g., grass, trees) on local climatic conditions and human physiological thermal comfort at pedestrian level.

## 2. Background and Study Area

### 2.1. The Challenges of Bilbao Municipality

Health impacts can be a concern in Bilbao, in northern Spain, where heat-related mortality is epidemiologically observed when the daily maximum temperature increases beyond 30 °C, around the 88th percentile of the daily maximum temperature of the summer months, suggesting a low level of population acclimatization to heat [62]. Under the expected high climate change scenarios (i.e., RCP8.5), heat wave events in Bilbao are expected to increase significantly in frequency, duration and intensity, resulting in substantially increased heat-related mortality in the absence of adaptation strategies [63,64]. As a complement to adequate heat warning systems, such as public health preventive interventions and health information plans, is crucial to develop long-term strategies like sustainable urban management and particularly green spaces, to reduce the population exposure to heat [65]. In this sense, the municipality of Bilbao is relying on improving the quality of urban and sub-urban green infrastructures through a new General Masterplan [66]. This practice, that is not always straightforward among the municipalities [67], aims to strengthen the greening systems of the city with the presence of vegetation elements (i.e., tree lined street and grass) which allow improving the human thermal comfort, the accessibility and the quality in the public spaces.

### 2.2. The Climate in Metropolitan Area of Bilbao of the Risk of Heat Wave in Basque Country

The Gran Bilbao metropolitan area hosts around 1 million inhabitants of which more than 340,000 live in the urban area of Bilbao municipality spread over an area of around 16 km^2^ [68]. Bilbao (latitude 43.25° N, longitude 2.96° W), is characterized by medium-high urban density and it is surrounded by a complex topography which has always affected the urban development of the city and its climate. The climate is humid temperate, with the absence of a dry season and a moderate level of temperature and precipitation during the year (Cfb Oceanic climate according to the Köppen-Geiger climate classification [69]). The highest annual solar radiation is registered in July, when the global horizontal radiation reaches up to 930 W/m^2^ as maximum hourly value during the day and more than 4790 W/m^2^ as the highest average daily value in a month. The level of RH achieves values higher than 70% during the entire year (above 75% in winter). In summer, the air T_a_ can surpass 30 °C from July until September [70]. The data registered by the Euskalmet [71] during the past decades show that the T_a_ has exceeded repeatedly 40 °C during summertime. The increment of T_a_ in *Gran Bilbao* area is also confirmed by González-Aparicio and Hidalgo [72]. Their statistical analysis has demonstrated that the heat wave events have affected significantly the people living in urban and sub-urban area of the Basque Country in the last two decades; and in the near future, the magnitude and the frequency of these events will significantly increase both in summer and in winter. According to the future projections of this study [72]. Regarding the warm season, the projections show an expected increment of T_a_ up to 3.5 °C in comparison to previous period (1978–2000) and an increase from 15 days (1978–2000) to 24 days (2020–2050) of total number of days for a single heat wave event [73]. This data is aligned with the predictions in other European studies [74,75,76,77]. In Bilbao, UHI temporal and spatial variations have been also studied [78]. Among other aspects, the airflow patterns, the complexity of the orography and the characteristics of urban morphology (e.g., geometric average of building heights, ratio of building plan area to total plan area, etc.) have a relevant influence in the variations of UHI magnitudes. The hereby-presented study aims to address green planning recommendations for urban decision makers to mitigate thermal stress and impact of future heat wave events inside typical urban canyons in Bilbao.

## 3. Methods and Materials

### 3.1. The Urban Case Study Areas

In line with other studies [73,79], main parameters, such as the height of the building (i.e., geometric average of building heights), the presence of vegetation, the building surface fraction (i.e., ratio of building plan area to total plan area) were used to select the study areas/districts (Table 1). The urban morphology analysis was conducted to extract the geometric dimensions of the selected districts and the characteristics of the local climate. Two main urban geometric aspects were considered: (1) the building surface area, i.e., the ratio between the surfaces covered by buildings (B) and the total surface (T), known as district’s urban density (B/T), and (2) the average values of the buildings’ height (H) and the streets’ width (W), known as urban canyon’s aspect ratio (H/W) (Table 1). All the measurements were taken from the cadastral virtual office of Biscay [80].

### 3.2. Microscale Numerical Modeling of ENVI-met

In this study, ENVI-met *v4* [81,82,83], was used to evaluate the evolution of thermal comfort within the urban street canyon. ENVI-met is usually adopted to analyze the interactions surface–plant–air for microclimate analyses for typical horizontal resolution from 0.5 to 10 m, and a period of 24–48 h. For each time step, the atmospheric equations solved by ENVI-met produce output data of typical meteorological parameters, such as T_a_, RH, T_mrt_, T_s_, W_s_ and wind direction (W_d_), radiation fluxes (Grad). ENVI-met v4 allows forcing air temperature and relative humidity to consider their evolution along the day and consequently the evaluation of thermal comfort conditions along the diurnal cycle. In this study, the evolution of hourly meteorological data along the 7th of August was set as the background airflow characteristics forcing in the model with the aim of representing summertime conditions in Bilbao and the characteristics of thermal stress levels in the urban area. All scenarios were run with the same boundary conditions to allow an adequate comparison between them. Data was taken from the meteorological station of Deusto (a station of the Basque Meteorological Network), located at latitude 4.28° N, longitude 2.93° W in the northern urban area of Bilbao at 3 meters above sea level [71]. The other meteorological data (i.e., W_s_, cloud cover, etc.) were set constant and are described in Section 3.5.

### 3.3. The Thermal Comfort Index of PET

The PET is based on the Munich Energy-balance Model for Individuals (MEMI) [6], which simulates the thermal conditions of the human body (Table 2).

PET is defined as the physiologically equivalent temperature and is equivalent to the air temperature at which, in a typical indoor setting, the heat balance of the human body is maintained with core and skin temperatures equal to those under the conditions being assessed. This way PET enables a layperson to compare the integral effects of complex thermal conditions outside with his or her own experience indoors [6]. It expresses the human thermal comfort in both indoor and outdoor environments using the international standard unit widely known as the Celsius degree (°C) [2]. This makes PET to be fully comprehensible by all the actors involved in the design process with or without technical background. PET gives the measure of thermal comfort considering the meteorological parameters: T_a_, RH, W_s_, T_mrt_. It also takes into account the physics of the human body: gender, height, activity, and clothing resistance for heat transfer, short-wave albedo and long-wave radiation of the surface affected by the physical surface properties [6,20]. In order to classify cold, neutral and heat stress in the urban canopies, the calculated values have been referred to the evaluation scale of Matzarakis et al. [5], that allows estimating the level of human thermal perception based on seven classes defined by [85] for Central Europe.

### 3.4. Measurement Campaigns

Measurements to validate the model performance were carried out during the summertime period in 2011 in an E-W oriented street. The width of the street was 24 m and the aspect ratio was mostly close to 1.0 in all the street canyon. The area is classified as compact midrise [78] although the street aspect ratio is slightly lower than in the selected urban areas (Section 3.1). The figure in Table 3 shows the location (red dot) of the measurements inside the area that was modelled to validate ENVI-met for the purpose of this study. The selection of the point was done aiming to consider a representative value in the street (i.e., sufficiently separated from the building facade and way from street intersections). T_a_, RH, W_S_, wind direction (W_D_) were measured at 8.5 m a.g.l. with a WXT520 Weather Transmitter (Vaisala,), mounted on a meteorological mast. Sampling was done every 5 seconds and 1-min average values were stored during the measuring period. The sensors provided the following accuracy for the measurements: ±0.3 °C for T_a_, ±3% for RH, and ±3% for W_S_ and W_D_. Additionally, 10-min average data of W_S_, RH, T_a_ and total incoming radiation measured in Deusto were used to evaluate meteorological boundary conditions during the measurement campaigns. The site is located in an open area surrounded by water 3 kilometres far from the measurements and validation area. More information can be found in Acero and Herranz-Pascual [86].

#### 3.4.1. Modelled Domain for Validation

**Figure 1:**
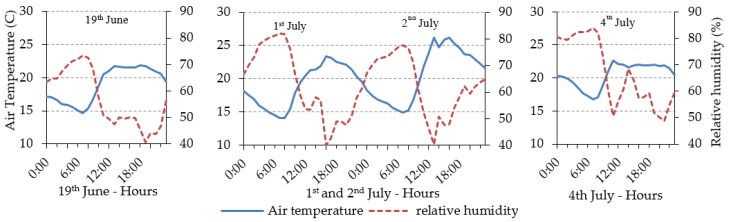
Hourly evolution of air temperature and relative humidity (forcing conditions for the model), measured at the Deusto station.

The domain used to validate ENVI-met covered an area of 300 × 300 m. The building characteristics were obtained from the Regional Government. The wall and roof material properties of the buildings are presented in Table 3. The vertical and horizontal resolution of all the model domain was 2 meters as has been set in previous studies [87]. The number of grid cells in the x, y, z directions were 150, 150 and 31, respectively. The size of the vertical grid cells was constant up to 26 m and then increased with height with a factor of 12%. Surface roughness was set to 0.2 m in correspondence with the surrounding area where the boundary conditions were measured. No specific vegetation elements were defined in the model due to the small size and low number of these. Their influence on the local microclimate is expected to be negligible. The surface materials were classified as light concrete for the pedestrian areas and asphalt for the traffic lanes. For the purpose of validating ENVI-met a few days of measurements (19th June, 1st July, 2nd July and 4th July) were selected with similar meteorological conditions to the ones used to analyse urban design scenarios in the current study (Section 3.5). This assures that the model was validated for the study’s purpose. Hourly evolution of T_a_ and RH, in Deusto (Figure 1) were used to force the model.

Each day W_S_ and W_D_ was considered constant, and the median value of the hourly data between 10:00 and 20:00 (UTC) was used as input to the model. Between these hours air flow characteristics registered in Deusto (i.e., 30 m a.g.l.) were quite similar and corresponded to a well-established sea breeze. W_S_ was adjusted to 10 m a.g.l. using the power law for wind profile [88,89]:

$$W_{S}{}_{10}=W_{S}{\left(10/h\right)}^{\propto }$$

where W_S_ is the wind speed (ms^−1^) at the height of h and α is an empirical exponent which depends on the surface roughness. In this case α = 1/7 because the measurements were done in an open terrain with obstacles not closer than 75 m. The estimation of solar radiation made by the model was adjusted by comparing with data measured at the Deusto station. The solar factor selected to adjust the shortwave solar radiation simulated by the model for each modelling period (i.e., each day) was at the maximum solar radiation hour (i.e., 12.00 UTC). Due to lack of information of soil moisture and temperature initial conditions, model default values were used. The meteorological input parameters are shown in Table 4. Simulations were launched at 4:00 local time (i.e., UTC+2), approximately 3 h before sunrise. The total modelling time was 44 h to allow the spin-up of the model. The last 24 h output (complete daily cycle) were considered for the analysis. A discrete receptor was defined inside the model to specify the location of measurements.

#### 3.4.2. Model Evaluation

The model validation was done by comparing hourly average measured data with ENVI-met results of T_a_, vapour pressure (*e*), and W_S_. Comparison was made with ENVI-met output values at 9 m height (the closest output to the measurements’ height).

**Figure 2:**
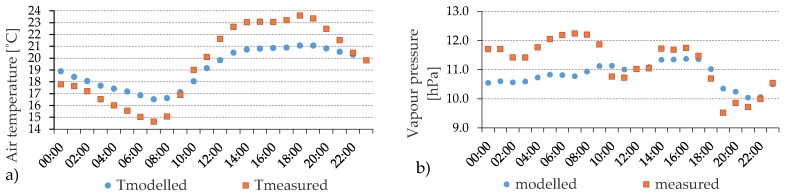
Hourly evolution of measured and modelled T_a_ (**a**) and e (**b**) on the 19th June 2011.

Figure 2 shows the results for 19th June 2011. Both the modelled and measured T_a_ and *e* describe a similar pattern. However, the modelled T_a_ shows lower values during daytime and higher values during the nighttime period. These results are related to the accumulation of heat inside the urban area and the boundary conditions that are forcing the model. During nighttime water surrounding the area from where boundary conditions are taken allows higher T_a_ (and thus modelled T_a_) than inside the street canyon (measured T_a_). Daytime results for *e* show a good agreement. However, model underestimation during nighttime can be associated with boundary soil moisture conditions. During the other days included in the model’s validation (1st July, 2nd July and 4th July), similar diurnal patterns of modelled and measured values are encountered.

To evaluate the relationship between modelled and measured variables correlation analyses were performed for the four days considered in the model validation.

The best relationship between modelled and measured data is for T_a_ and *e* with Pearson correlation coefficients of 0.951 (*p* < 0.001) and 0.827 (*p* < 0.001) respectively. The accuracy of ENVI-met simulations (i.e., the degree to which modelled values approach measured values) was evaluated using the root mean squared error (RMSE), the mean absolute error (MAE) to summarize the difference between modelled and measured variables, and the dimensionless index of agreement (*d*), a descriptive measure to make cross-comparisons between models outputs [90]. Results are shown on Table 5. RMSE (1.87 °C) and MAE (1.64 °C) for T_a_ are close to the median value of reported values of RMSE (1.51 °C) and MAE (1.34 °C) in other ENVI-met studies [91,92]. A study in a close by area of Bilbao provided similar RMSE values, between 1.00 and 2.07 °C, and MAE values, between 0.83 and 1.82 °C [93].

Another study in Phoenix (Arizona, USA) also showed a range of values between 1.41 °C and 2.00 °C for RMSE and between 1.18 °C and 1.74 °C for MAE depending on the land use [94]. In the case of *e* and W_S_, RMSE values correspond to 9.5% and 165.9% and MAE values correspond to 8.0% and 149.4% of the mean measured value during the 4 days. The results for W_S_ are influenced by the limitations of the model to be forced with changing airflow conditions. Results improve when comparing the pairs (measured & modelled) under the same airflow condition [i.e., sea breeze; between 10:00 and 20:00 (UTC)], but still RMSE (1.28 m/s) and MAE (1.14 m/s) are high representing 104.6% and 89.6% respectively of the mean measured value. Similarly, the *d* for W_S_ is low showing the limitations of the model to represent correctly the measured values. However, results are in accordance with previous studies [82,93,95,96].

On the contrary, T_a_ and *e* present high *d* values similar or even higher than the ones reported in other studies with ENVI-met [91,93], representing a suitable performance of the model. From the results of the model validation, it is concluded that ENVI-met simulations have a deviation with respect to measurements taken in the area used for model validation (compact midrise). Although RMSE, MAE and *d* values obtained for T_a_ and *e* can be consider reasonable and are similar to other works with ENVI-met available in literature [82,93,95,96], the model lacks a validation for T_mrt_ (crucial parameter for thermal comfort evaluation) that is a limitation of the study (see Section 4.6).

### 3.5. Model Settings for Scenarios Analysis

Each spatial domain, modelled in ENVI-met environment, was constituted by four blocks and three UCs. The typical geometric proportions of each UC of the selected urban areas (Table 1) and the vegetation elements (i.e., tree, grass) have been reproduced, while the size of the grid cells (2–3 m, Table 6) was set according to the width of the street so as to provide reliable results. ENVI-met projects each of vegetation element to the corresponding grid cells in domain, varying their climatic properties (e.g., humidity and roughness).

The nesting grids were set equal to 7. As recommended by Bruse [97], the spatial domain along the z-direction was set at least twice the height of the tallest building. The length of the buildings’ blocks was set equal to six times of their height [98] to avoid any perturbation from the borders. 

In order to consider the city’s surrounding, the roughness of the urban environment and the material of the soil outside the area of the model was set accordingly. The buildings blocks were modelled as completely straight volumes to avoid any obstacle, barrier and obstruction (i.e., shelters, balconies, decorative items on the façades and roofs). 

**Figure 3:**
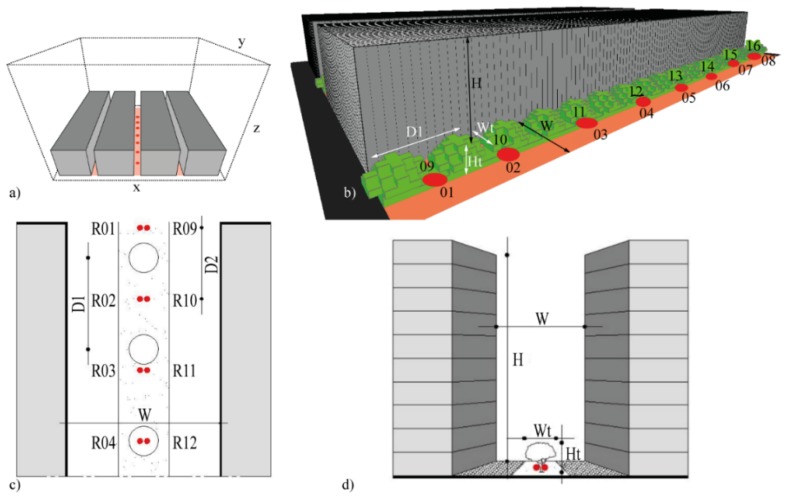
3D model of the four blocks and three UCs in the spatial (x, y, z) domain (**a**), view of a street (**b**), horizontal (**c**) and vertical (**d**) sections. The receptors (red points), trees’ location and distance between trees (D_1_) and receptors (D_2_) are visualized.

The materials were set to reproduce the real materials of walls, roofs and soil, which are typically used in the analyzed urban areas (Table 7). For the green scenarios, the central part of the street canyon was with grass, covering 30% of the total street’s width. Grass had a height of 0.1 m. The foliage of the trees’ crown was set equal to 2/3 of full-fill density (Table 8). The distance between the aligned trees (D1) was set to maintain a constant ratio of foliage coverage (D1/Wt) in all scenarios (Figure 3).

Fourteen specific receptors were positioned in the central part of the urban canyon at equidistant from each other (D2). The receptors allowed assessing the thermal comfort using PET index [15]. Modelled results were saved every 30 min. Data was analyzed at 1 m above the ground surface except for the surface temperature, calculated at z-Grid = 0.

The spatial distribution of the receptors was set to study the local benefits given by the presence of the trees: some receptors (e.g., R02, R09) were located between the trees, others (e.g., R04, R12) under the trees’ crown. All model settings for building blocks were set to generalize the geometry of the analyzed urban areas and facilitate the replicability of the methodology in different parts of the city where geometric characteristics (H/W and B/T) and presence of vegetation elements are similar.

The start time of the simulations was stated at 4:00 a.m. of the 6th of August local time (i.e., UTC+2), approximately 3 h before sunrise, while the total modelling time was set as 44 h. The first 20 h are necessary to spin up the model [99], and therefore, only the outputs related to the last 24 h (i.e., from 0:00 to 24:00 of the 7th of August) were considered (Table 6).

### 3.6. Scenarios

The comparative analysis of human thermal comfort was conducted in four scenarios. In the first scenario, the typical orientation of the selected urban areas was considered. This part, named as scenario S0, focuses on the outdoor human thermal comfort at 1 m high from the ground level in the selected areas characterized by the typical orientations: 24° North-South (N-S) in Casco Viejo; 17° N-S in Abando Indautxu; and 9° N-S in Txurdinaga/Miribilla (Table 9). In the second set of simulations, four standardized orientations such as N-S, East-West (E-W), North/East-South/West (NE-SW) and South /East-North/West (SE-NW) for all the selected urban areas and different pavement materials of the street were analyzed in all the analyzed urban areas. This analysis, named as scenario S1, aims to study the effect of street pavement material when changing from asphalt (albedo = 0.12), to pedestrian boulevards with red brick stone (albedo = 0.30). In Bilbao these interventions, which started during the last decade to promote new urban public spaces, are more frequent in compact mid-rise and open-set high-rise urban areas. Differently, in compact low-rise urban area, such as the historic center of Casco Viejo, the traffic was highly limited, therefore the decorative red brick stones were originally used as pavement material. In all scenarios no vegetation elements were set (Table 9).

In the last set of simulations, the mitigation strategies using vegetation elements were studied. Two mitigation scenarios through urban green interventions without giving any obligation or disposition to dwellings’ owners and designers to plan any intervention at building level were analyzed. The scenarios are characterized by a loamy ground soil positioned in the central part and covering 30% of the street’s width, while the rest of the street was covered with red brick stones. The loamy soil included the presence of grass, while a tree-lined was set in the central part of the street. These features were set in all urban areas as follows:

(i) Mitigation scenario 01 (M01): the height of the trees (Ht) was set proportionally to the height (H) of the analyzed urban canyons, by maintaining constant the ratio Ht/H = 0.25 (Table 10);
(ii) Mitigation scenario 02 (M02): beyond maintaining constant the ratio Ht/H = 0.25 also the width of the trees (Wt) was set proportionally to the width (W) of the analyzed urban canyons, by maintaining constant the ratios Wt/W = 0.3 (Table 10).

## 4. Results and Discussion

### 4.1. The Effect of the Orientation

**Figure 4:**
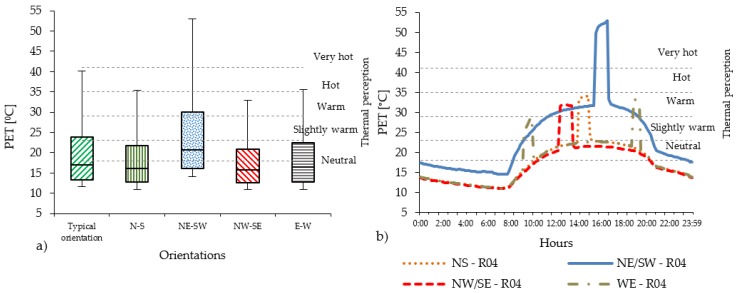
(**a**) PET values measured in all 14 receptors within the UC with brick pavement for compact low-rise urban areas; (**b**) The PET hourly evolution in R04 of PET in compact low-rise urban canyon.

In the compact low-rise urban areas, the level of PET varies substantially with the orientations. Considering all 14 receptors (Figure 4a), the PET level in Casco Viejo generally results quite high. N-S and E-W orientations are mostly below the hot thermal perception range (35 °C < PET < 41 °C), while for the NE-SW orientation, the PET value reaches the very hot thermal perception level (PET > 41 °C). The NW-SE orientation shows slightly lower PET level than the N-S, E-W orientation. Regarding the diurnal cycle (Figure 4b), PET peaks are distributed in different moments of the day according to the streets’ orientation: around midday for N-S orientation; early in the morning and evening for E-W orientation. Out of the peak periods, the PET level remains in the neutral level in all orientations, except for NE-SW orientation. 

**Figure 5:**
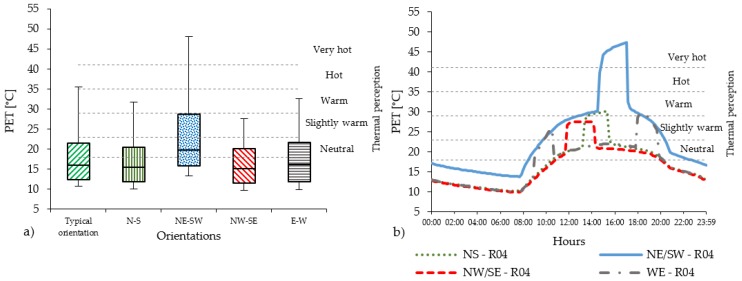
(**a**) PET values measured in all 14 receptors within the UC with asphalt pavement for compact mid-rise urban areas; (**b**) The PET hourly evolution in R04 in the compact mid-rise UC.

NE-SW orientation has the highest peak values, around 20 °C higher than the rest of the orientations (i.e., two thermo-physiological stress classes). In the compact mid-rise urban areas, the peak levels of PET reach the range of moderate heat st ress (29 °C < PET < 35 °C) for the N-S and W-E orientations, which is one thermophysiological class less than the existing typical orientation of Abando/Indautxu (Figure 5a); while for NW-SE orientation, the peak levels are in the range of slightly warm thermal perception (23 °C < PET < 29 °C) (Figure 5b).

**Figure 6:**
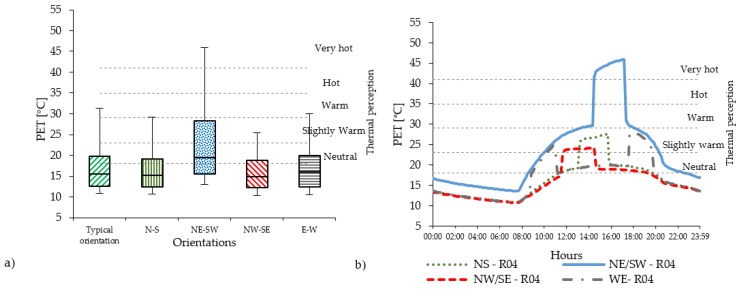
(**a**) PET values measured in all 14 receptors within the UC with asphalt pavement for the open-set high-rise urban areas; (**b**) The PET hourly evolution in R04 in the open-set high-rise UC.

The diurnal cycle shows that the intensity of PET remains within the range of neutral thermal stress for N-S, NW-SE and E-W orientations, while the NE-SW orientation has the highest PET levels. In the open-set high-rise urban areas there is a relevant reduction of the PET peaks (Figure 6a) due to the geometry of the urban canyon (i.e., low aspect ratios H/W). The distribution and the duration of PET peak also change considerably (Figure 6b) (see Section 4.2).

### 4.2. The Effect of Streets’ Orientation on the Duration of PET’s Peak

**Figure 7:**
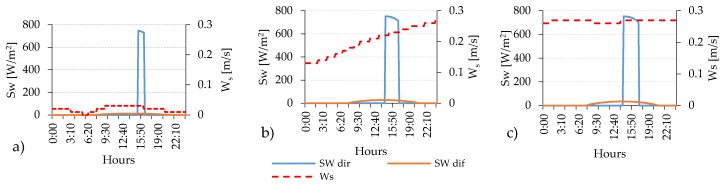
The hourly evolution of the typical trend (R04) of short-wave direct (Sw dir) and diffuse (Sw dif) irradiation and the W_s_ at pedestrian level within the (**a**) compact low-rise, (**b**) compact mid-rise and (**c**) open-set high-rise urban canyons.

The analyses for standard orientations have confirmed that the best performing orientation in terms of thermal comfort standards is the NW–SE one where the PET level remains in the range of neutral thermal stress for the major part of the day, except during the peak period that it remains within the warm thermal sensation in all urban areas (Figure 4a, Figure 5a and Figure 6a). The worst performing orientation in terms of thermal comfort standards is the NE-SW, where the average PET level stays under the warm thermal stress limit (PET < 29 °C) in the open-set high-rise and compact mid-rise urban area, while only in the compact low-rise that limit is overcome. The duration of the peak period varies from 1 h for compact low-rise urban canyon, where the PET level reaches almost 53 °C, to more than 2 h and 30 min in compact mid-rise and open-set high-rise urban areas, in which the PET value is more than 45 °C. For the best orientation (NW-SE) the duration of the peak period coincides with the duration of the thermal discomfort period (PET > 23 °C) while for the NE-SW orientation, the thermal discomfort persists for over 10 h in all urban canyons (Table 11). These aspects are affected by the orientation and the aspect ratio of each urban canyon, which impact on the presence of direct solar radiation and the level of W_S_. In fact, even if the short-wave (Sw) direct irradiation remains over 745 W/m^2^ during the peak in all urban areas, the average wind speed presents significant differences with 3.74 m/s in open-set high-rise, 2.74 m/s in compact mid-rise against 1.3 m/s in the compact low-rise urban areas (Figure 7 and Table 11).

### 4.3. Impact of Pavement Materials

**Figure 8:**
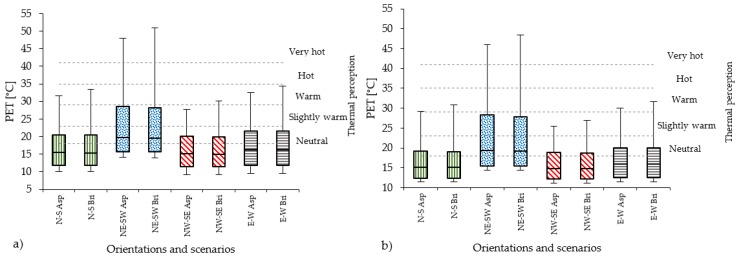
PET values measured in all 14 receptors for scenarios with asphalt (Asp) and brick red stones (Bri) pavement for compact mid-rise (**a**) and open-set high-rise (**b**) urban areas in all orientations.

The reconversion of the street from vehicular traffic with asphalt pavement to pedestrian promenade with pavement in red brick stones, especially changes in T_s_ and T_mrt_ are observed. Overall, these cause an increase in PET levels in all orientations (Figure 8). The highest value of T_s_ with asphalt pavement reaches 44.1 °C in the compact mid-rise and 40.1 °C in open-set high-rise areas, while with red brick stones it is reduced to 37.8 °C and 38.5 °C in the compact mid-rise and open-set high-rise areas, respectively. T_mrt_ values surpass 60 °C in all orientations with both asphalt and red brick stones, while the highest values, over 70 °C, persist for NE-SW. In all urban typologies, T_mrt_ is generally higher with red brick stone. In the case of T_a_ the differences between asphalt and red brick stones are lower than 5 % and 3 % for compact mid-rise and open-set high-rise urban areas, respectively. W_s_ and RH show the same values for both pavement types. Regarding PET, red brick stones increase the thermal stress at pedestrian level in all street orientations, although the thermophysiological class rises from slightly heat stress to moderate heat perception only in the compact mid-rise urban canyon for NW-SE orientation and in the open-set high-rise for N-S orientation (Figure 8 and Table 12).

### 4.4. Spatial Differences Inside the Street Canyon

**Figure 9:**
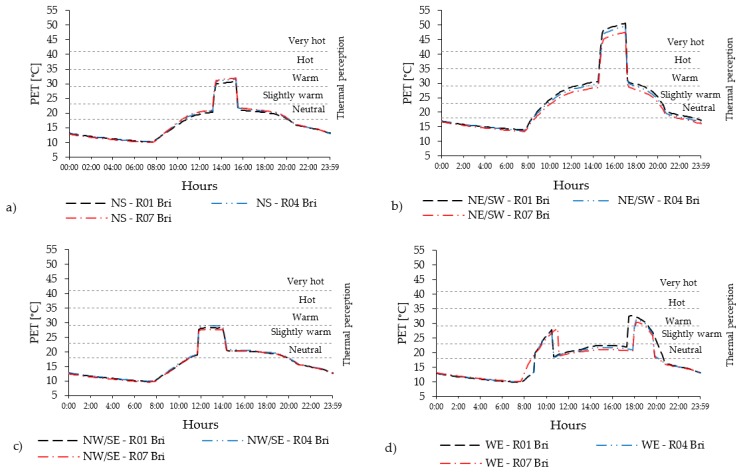
Spatial differences inside the UC of compact mid-rise urban and pavement in red brick stones: hourly evolution of the PET for different orientations (**a**) NS, (**b**) NE/SW, (**c**) NW/SE and (**d**) WE for receptors located at the beginning (R01) in the middle (R04) and at the end (R07).

Another relevant effect is the PET evolution along urban canyons. In both compact mid-rise and open-set high-rise areas, the PET is affected locally: differences between 1 and 1.5 °C values of PET are registered at the beginning (R01), in the middle (R04) and at the end (R07) of the urban canyon. For example, for the W-E orientation, R01 is under the Sun for a longer time than for R04 because it is localized in a point that is not affected by the buildings’ shadows in the early morning and in the late afternoon (Figure 9d).

**Figure 10:**
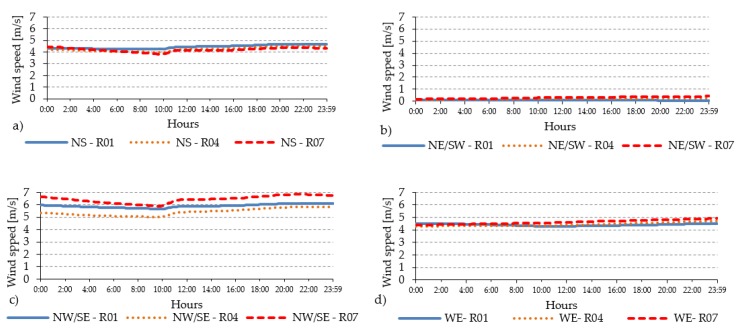
Spatial differences inside the UC of the compact mid-rise urban: the hourly evolution of the W_s_ for different orientations (**a**) NS; (**b**) NE/SW; (**c**) NW/SE and (**d**) WE for R01, R04) and at R07.

In addition, the daily profile of W_S_ is different along the urban canyon. The highest spatial differences along the street canyon occur for NW-SE orientation (Figure 10) which corresponds with the prevalent wind direction in Bilbao (used as boundary meteorological condition in the simulations). In all orientations, the highest W_S_ values occur at the beginning and at the end of the canyon characterized by an airflow “channelling effect”.

### 4.5. Impact of Vegetation Elements

The PET peak values in the mitigation scenarios M01 (Ht/H = 0.25, grass and trees) and M02 (Ht/H = 0.25, Wt/W = 0.3, grass and trees) show that the highest thermal stress is always for the NE-SW orientation. For this orientation, the heat stress remains at the extreme level (PET > 41 °C) in the compact low-rise and compact mid-rise urban areas, while in the open-set high-rise the PET results just below the 41 °C (i.e., in the strong heat stress range) (Table 13). 

The lowest PET peak values are reached in the NW-SE orientation in all urban areas in correspondence with the predominant wind conditions in Bilbao. For this orientation, the PET value varies from warm level in compact low-rise urban areas (31.6 °C in M01 and 30.6 °C in M02) to slightly warm level in compact mid-rise (27.4 °C in M01 and 26.4 °C in M02) and open-set high-rise urban areas (27.0 °C in M01 and 25.5 °C in M02).As has been demonstrated in previous studies [100,101,102,103,104,105], the presence of trees has a limited and localized benefit, highly dependent on the reduction of incoming solar direct radiation given by the shadow of the trees’ crown. Relevant local benefits are due to the presence of shadow created by the geometry (i.e., height, shape and width) and the foliage density of the trees’ crown, the buildings’ geometry and the urban canyons’ orientation. In all urban canyons and orientations, the highest PET values are registered at the beginning and the end of the urban canyon due to the low shadow of buildings and trees’ crown during the day, as happens in R01 and R07, which are not localized in proximity or under a tree (Figure 3).

#### 4.5.1. Impact of Mitigation Effect in the Compact Low-Rise Urban Areas

**Figure 11:**
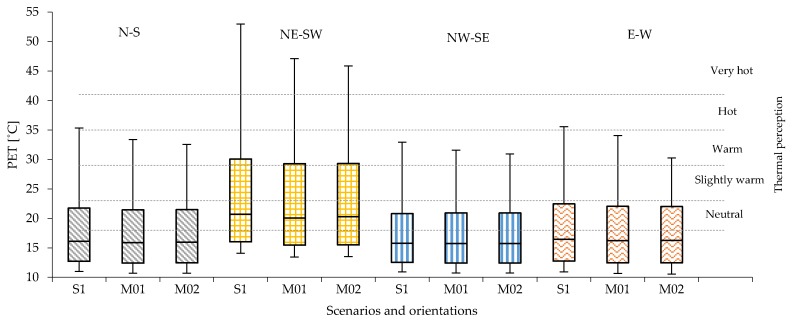
PET values measured in all 14 receptors in compact low-rise urban areas for scenario S1 with red brick stones and mitigation scenarios M01 (Ht/H = 0.25) and M02 (Ht/H = 0.25 and Wt/W = 0.30) in all orientations.

In the compact low-rise urban area, the presence of grass and trees improves the PET peak up to one thermophysiological level (Figure 11).

Slightly higher reductions are expected in the NE-SW and E-W orientations. Along the urban canyon, relevant local differences in PET peak values can be encountered. In scenario M01, N-S and E-W orientations show the highest differences (5 °C) between R01, R04, R07, while scenario M02 is the NE-SW orientation that reaches 4.6 °C between the three points. In both M01 and M02, the highest reduction is registered in the NE-SW orientation for receptor R04 (middle of the street canyon). In all street orientations and locations inside the street canyons (receptors), reductions in PET peak is always higher in M02 (Table 14). The daily evolution of PET in R04 in the NE-SW orientation shows that, despite in the scenario M01 the value of PET decreases about 11.3 °C with respect to the scenario S1 without vegetation, the thermal stress remains above the limit of a very hot level (PET up to 41.7 °C).

**Figure 12:**
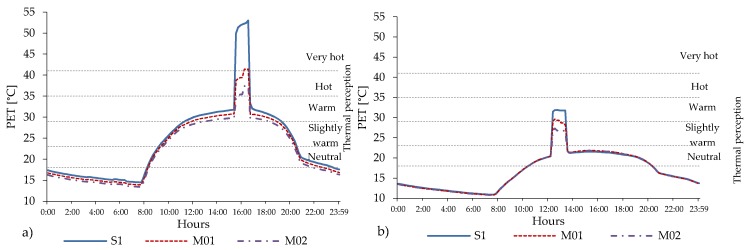
Daily evolution of the PET level in compact low-rise urban area for scenarios S1, M01 and M02 registered in R04, for the worst orientation NE-SW (**a**) and the best orientation NW-SE (**b**).

The cooling benefit given by the presence of trees and grass is more perceivable in M02, where the PET level reaches 37.7 °C (Figure 12). Less improvement is reached in the NW-SE orientation, in which the PET level decrease about 9.2 °C for scenario M02 from warm to comfortable thermal stress sensation class. The PET peak lasts for one hour from 15:30 to 16:30, while the duration of the thermal discomfort (PET > 23 °C) lasts for 11 h in S1 and M01, while it is reduced by 20 min in M02.

#### 4.5.2. Impact of Mitigation Effect in the Compact Mid-Rise Urban Areas

**Figure 13:**
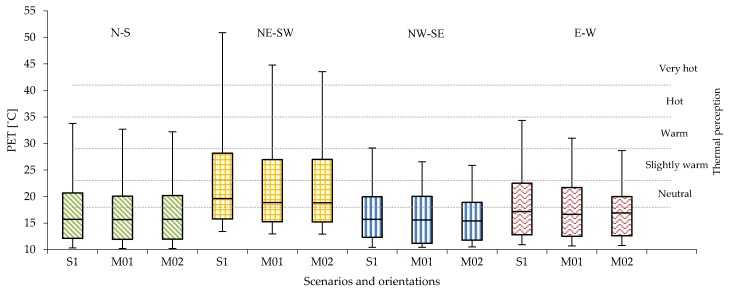
PET values registered in all 14 receptors in the compact mid-rise urban areas for scenario S1, M01 and M02 in all orientations.

The data for the mid-rise urban areas confirms that the combined presence of grass and trees generally improve the human thermal comfort along the entire urban canyon in all orientations about one thermophysiological level of PET (Figure 13).

In the NE-SW orientation, the presence of trees in M01 and M02 provide a local benefit along the canyon by reducing PET peak from very hot thermal heat stress. The PET overcomes the threshold of 41 °C in S1 to the range of hot level of thermal stress with PET equal to 39.0 °C in M01 and to 37.0 °C in R04 and to 36.5 °C in R07 in M02 (Table 15). In NW-SE orientation, the cooling benefit is higher by reaching two thermophysiological classes: passing from warm thermal heat stress with PET level of 29.1 °C in S1, to slightly warm in M01 and PET equal to 23.9 °C until comfortable thermal heat stress in M02 with a PET value of 21.4 in R04 and of 20.8 in R07 (Table 15).

**Figure 14:**
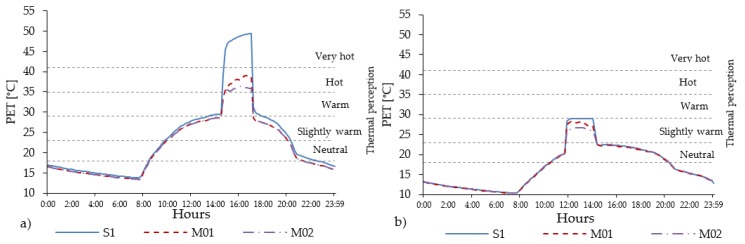
Daily evolution of the PET level in compact mid-rise urban area for scenarios S1, M01 and M02 registered in R04, located in the middle of the urban canyon for the worst orientation NE-SW (**a**) and the best orientation NW-SE (**b**).

The daily evolution of PET level in R04 shows that intensity of the peak values lasts for more than two hours: from 14:40 to 17:00, which results to be more than double in comparison to the compact low-rise urban areas (Figure 14).

Regarding the duration of intensity of peak value of PET, the different geometry of the trees in M02 contribute to reduce it up to 20 min. This effect is even stronger in relation to the duration of thermal discomfort (PET > 23 °C): in the M01, the reduction is about 20 min; while in the M02, the benefit given by the presence of the tree allows a decrease of more than one-hour compare to S1.

#### 4.5.3. Impact of Mitigation Effect in Open-Set High-Rise Urban Areas

**Figure 15:**
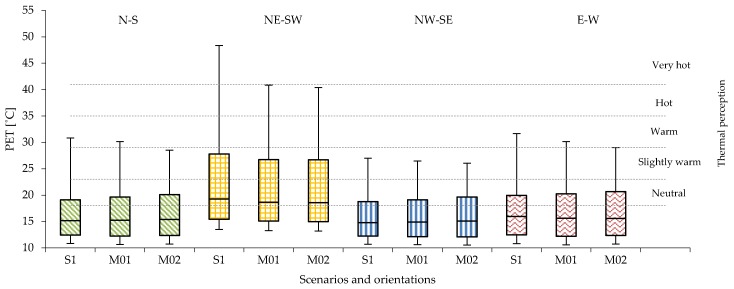
PET values registered in all 14 receptors in the open-set high-rise urban areas for scenario S1, M01 and M02 in all orientations.

**Figure 16:**
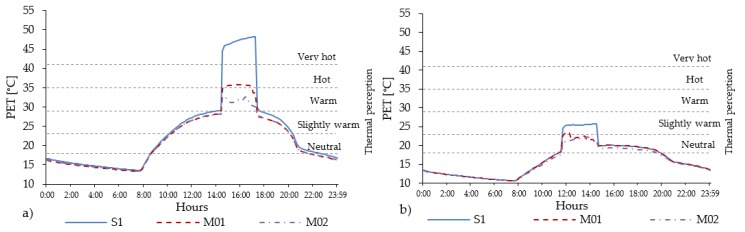
Evolution of PET level in open-set high-rise urban area for scenarios S1, M01 and M02 in R04, located in the middle of the UC for the worst NE-SW (**a**) and the best NW-SE (**b**) orientations.

The analyses conducted in the open-set high-rise urban areas confirm the effects observed in the other urban areas. However, in these urban areas the PET values are consistently lower (Figure 15) than in the other urban areas. The PET level in different points along the urban canyon with the combined presence of grass and trees can benefit up to two thermophysiological levels of PET in all orientations (Table 16). Figure 16 shows the daily evolution of the PET level in the middle of the urban canyon (R04) in the scenario S1 and in the mitigation scenarios M01 and M02 for the NE-SW orientation, for which the highest value of thermal heat stress has been registered and for the NW-SE orientation, characterized by the lowest thermal stress.

The highest intensity of PET reaches 48.2 °C and lasts for 2 h and 40 min, from 14:30 to 17:10. This is the longest heat stress period among the all analysed urban canyons. In terms of cooling effect given by the presence of vegetation elements, the highest PET reduction equal to 12.4 °C was observed in the scenario M01, while in M02 it reached 15.4 °C. These reductions allowed lowering the heat thermal stress from very hot thermophysiological class in scenario S1 to the hot and warm thermal stress levels in scenarios M01 and M02, respectively.

Regarding the duration of the thermal discomfort (PET > 23 °C), there are relevant differences among the different scenarios. In the scenario S1, the thermal discomfort period lasts for more than 10 h from 10:00 until 20:10, while the mitigation scenario M01 only reduces this period about 10 min (it ends at 20:00) and finally the mitigation M02 decreases this period about half an hour. This is due to the geometry of the trees’ crowns.

### 4.6. Limitation of the Study

It is worth describing some of the limitations of the current study. Firstly, thermal stress evaluation requires the measure of four physical (air temperature, mean radiant temperature, humidity and air velocity) and two subjective (metabolic rate and thermal clothing insulation) parameters [106]. Despite the fact that the climate model in ENVI-met has been validated for T_a_, RH, no validation was carried out for T_mrt_ due to the lack of measurements of this variable which could be considered a relevant limitation of the study. However, other studies in Bilbao [93] already evaluated this parameter showing a limitation of the model to represent correctly variations of incoming radiation (and thus, T_mrt_) during daytime. This constraint is less relevant for the current study where a comparison among different scenarios has been conducted under the same boundary conditions that represent a clear sky day in Bilbao. Also, the validation of the model was done for one reference point. Although further points could be checked/validated, the chosen one can be considered representative of a compact midrise street due to its location close to its middle and away from intersections. Moreover, the data are related to only one day at each UC.

Secondly, building facades have been modelled as opaque walls while most of them combine windows and concrete facades. In a subsequent study, detailed data on the real stratigraphy of the opaque walls and specific data on the transparent parts (windows) could be provided in order to improve the accuracy of the results and also evaluate the influence on outdoor thermal comfort of the window-to-wall ratio (WWR) [107].

Thirdly, the findings (although based on hypothetical/simple urban shapes) are valid for districts that have similar characteristics as the urban areas analyzed in this study. However, the same methodology could be replicated in different parts of the city with other geometric proportions such as a-symmetrical canyons, which effects have been treated in some studies [108,109].

Fourthly, the results have been analyzed for specific receptors and not for the whole space inside the street canyon where different outcomes, at some extent, can be expected. Despite the approach does not include a detailed spatial analysis, the results show clearly the effects of the different mitigation strategies in Bilbao. These could be included in the coming Master Plan of the city to improve thermal comfort levels and reduce the impact of heat waves.

Finally, the study has been carried out with the same boundary conditions for all the study areas which somehow guarantees consistency of the results, i.e., see the effect of the mitigation strategies in different urban developments. However, recent studies have found that better outcomes of ENVI-met can be expected when the model is forced with measurements inside the modelled domain [110,111]. Thus, probably a better comparison could have been possible if measurements in the three types of urban developments would have been available.

## 5. Conclusions and Further Developments

**Figure 17:**
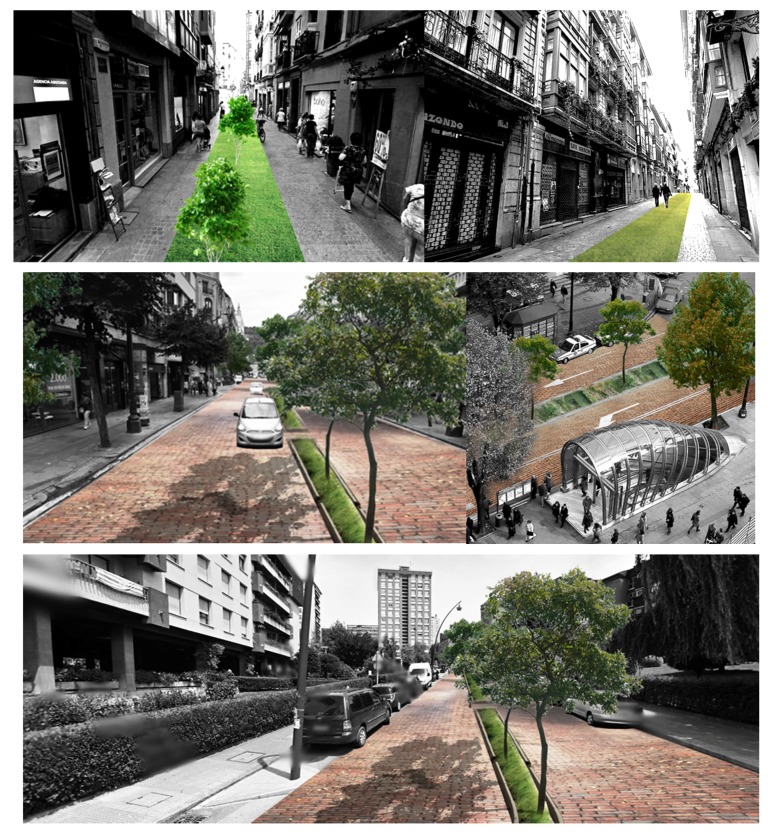
Visualizations of potential green intervention in compact low-rise Casco Viejo (top), compact mid-rise Abando/Indautxu (middle) and open-set high-rise Txurdinaga/Miribilla (bottom) urban areas of Bilbao. Elaboration from Google Street views.

This work presents the analysis conducted on a summer day in Bilbao to study the effect of different orientations (i.e., N-S, NE-SW, NW-SE, W-E), aspect ratio (H/W), pavement materials (i.e., asphalt and red brick stones) and vegetation elements (i.e., grass and trees) on human thermal stress at pedestrian level inside typical urban canyons of low-rise, mid-rise and open-set high-rise urban areas. The findings are presented as planning recommendations visualized in (Figure 17):

- Urban parameters such as aspect ratio and orientation were found to have a significant influence on the human thermal comfort at the pedestrian level. In all urban areas, for a NE–SW orientation the solar radiation has the highest impact on thermal discomfort. In open-set high-rise urban areas the presence of the trees could produce a relevant reduction in thermal stress at pedestrian level. Furthermore, orientation and aspect ratio have a considerable influence on the intensity of the PET peak, its duration and on the period of thermal discomfort (PET > 23 °C).
- The reduction of the intensity of the thermal stress at the pedestrian level and its spatial extent highly depend on the vegetative measures applied inside typical urban canyons. In the analyzed scenarios, the highest PET peak reduction due to tree-lined streets reaches 15.3 °C. Tree-lined streets composed of species with tall and broad crowns are more effective because of the large vegetation volume and leaf biomass inside the canyon. Similar to previous studies in Bilbao [100] the results show that the cooling effect provided by this arrangement of trees is in general locally restricted to the immediate vicinity of the trees.
- The benefit in terms of human thermal comfort created by the presence of the vegetation elements, is more significant in the proximity of the tree-lined streets. In that regard, for the R04, localized under a tree, the benefit reaches a reduction of up to two PET thermal perception classes (depending on the street orientation) in all urban areas.
- Regarding the thermal effect of pavement materials, it was demonstrated that replacing asphalt with decorative red brick stones reduces the surface temperature value, but increases the T_mrt_ and PET at the pedestrian level. Thus, materials used in pedestrian areas in Bilbao are not beneficial to reducing heat thermal stress.
- This study has demonstrated that street orientation, aspect ratio and the presence of vegetation consistently influence the wind speed at the pedestrian level and the cooling effect provided by street ventilation. Therefore, municipalities should adequately choose the types of trees to plant in relation to the urban canyon geometry.

The methodology used for this study can be applied in the early planning stages to support urban planners and decision makers to reduce the risk of human thermal stress inside urban canyons and districts. In fact, conducting quantitative and qualitative analyses allow evaluating and considering which urban interventions should be prioritized in new and/or in consolidated urban areas in order to guarantee thermal comfort at the pedestrian level. Future development of the study could include an economic evaluation to estimate the financial impact of each specific intervention and its relation with indoor energy consumption. Furthermore, analyses on the use of novel cooling materials for pavement such as retro-reflective could be undertaken [112,113,114]. Finally, another potential intervention in terms of cooling effect can be the application of vegetation elements on the building envelope such as green façades on existing and new buildings [115,116].

## Figures and Tables

**Table 1:** Analysis of the current situation of the selected urban case study areas.

Data ^1^	Compact Low-Rise	Compact Mid-Rise	Open-Set High-Rise
**a)**	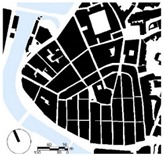	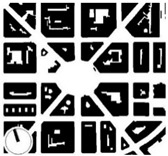	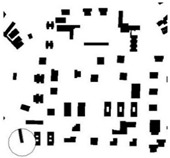
Selected urban areas	Casco Viejo	Abando/Indautxu	Txurdinaga/Miribilla
Type of area	Historic, residential and commercial	Business center, residential and commercial	Residential and service
T—Total area [m^2^]	175,000	890,000	180,000
B—Built area [m^2^]	140,000	360,000	72,000
Urban density [B/T]	B/T > 0.60 [0.8]	0.40 < B/T ≤ 0.60 [0.60]	B/T ≤ 0.40 [0.40]
**b)**	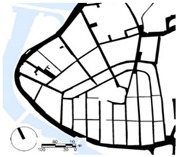	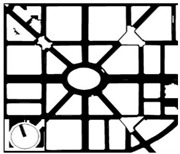	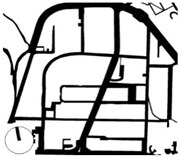
H—Buildings’ heights [m]	16 (4/6 floors—attached)	24 (7/10 floors—attached)	40 (>9 floors—single high rise buildings)
W—Streets’ width [m]	4.5 (narrow street)	16 (wider avenues of four traffic lanes)	30 (large avenues of two or more traffic lanes)
Aspect ratio [H/W]	H/W > 1.5 [3.5]	1.3 < H/W ≤ 1.5 [1.5]	H/W ≤ 1.3 [1.3]
**c)**	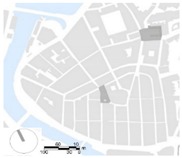	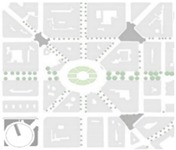	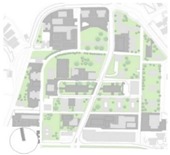
Total green areas [m^2^]	0 (None)	7500 (None-Low)	50,000 (None-Low)
Incidence green areas ^a^	0.00 %	0.85 %	20.0 %
Squares/void spaces [m^2^]	3500	40,000	2400
Incidence squares ^a^ [%]	2.0	4.5	1.3
Percentage occurrence ^b^	4.8 %	17.1 %	23.8 %
Façade materials	concrete/brick/stone	concrete/brick/stone	concrete/brick
Roof materials	terracotta	terracotta	terracotta
Type of soil	red brick stone	asphalt	asphalt

^1^ Source: [78]. ^a^ Ratio related to total area of the selected district in Bilbao and green spaces/squares presented in those areas. ^b^ Ratio of total land use category area to total urban area in Bilbao.

**Table 2:** PET level, thermal perception and grade of physiological stress according to [5,84]. On the right, a sample of heat balance calculation with the MEMI in summer (figure modified from [6].).

PET	Thermal Perception	Grade of Physiological Stress	Heat Balancing (MEMI): Summer
	Very cold	Extreme cold stress	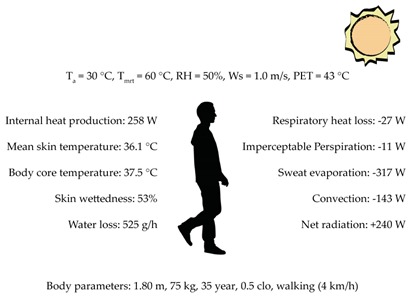
4 °C			
	Cold	Strong cold stress	
8 °C			
	Cool	Moderate cold stress	
13 °C			
	Slightly cold	Slight cold stress	
18 °C			
	Neutral	No thermal stress	
23 °C			
	Slightly warm	Slight heat stress	
29 °C			
	Warm	Moderate heat stress	
35 °C			
	Hot	Strong heat stress	
41 °C			
	Very hot	Extreme heat stress	

**Table 3:** Analysis of the current situation of the selected urban case study areas.

**Data**	**Walls**	**Roofs**	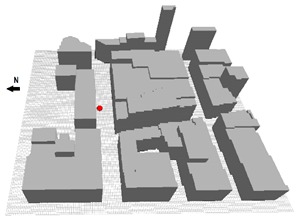
**Description**	Burned Brick	Tile	
**Thickness [m]**	0.25	0.20	
**U-value [W/m^2^K]**	0.44	0.84	
**Albedo**	0.40	0.50	
**Emissivity**	0.90	0.90	
**Specific heat [J/kgºC]**	650	800	

**Table 4:** Description of the model meteorological boundary configuration of each day of simulation.

Meteorological Variable	Days in 2011			
	19th June	1st July	2nd July	4th July
Wind Speed at 10 m a.g.l.	3.0 m/s	3.4 m/s	3.8 m/s	4.3 m/s
Wind direction	287º	312º	263º	308º
Cloud cover (oktas)	1	1	1	1
Specific humidity (2500 m)	4.0 g/kg	2.44 g/kg	3.54 g/kg	6.65 g/kg
Solar adjust Factor	0.84	0.87	0.84	0.84

**Table 5:** Quantitative difference metrics of modelled with respect to measured T_a_, *e*, W_S_ for the four days of comparison (19th June, 1st July, 2nd July and 4th July 2011).

Sample Size	Difference Measures			
		T_a_	*e*	W_S_
96	RMSE	1.87	1.22	1.55
	*MAE*	1.64	1.03	1.39
	*d*	0.89	0.87	0.35

**Table 6:** Input configuration data applied in the models to run the ENVI-met simulations.

**a) Initial Meteorological Conditions**							
Wind speed measured at 10 m height (m/s)				4.0			
Wind direction (deg)				315° (0° = from North … 180° = from South…)			
Roughness length at measurement site				0.2			
Specific humidity at model top (2500 mg/kg)				4.5			
Relative humidity at 2 m height (%)				63.3			
Forced values of air temperature and relative humidity							
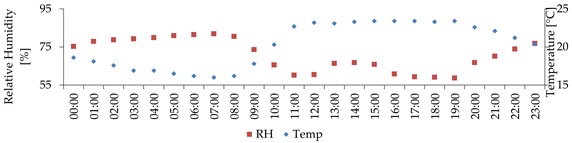							
**b) Solar radiation and clouds**							
Adjustment factor for solar radiation				0.86			
Cover of low clouds (octas)				1.00			
Cover of medium clouds				0.00			
Cover of high clouds				0.00			
**c) Soil data**							
Initial temperature in all layers: 0–0.2 m; 0.2–0.5 m; >0.5 m (°K)				293.4 (equivalent to 20.3 °C)			
Relative humidity upper layer (0–20 cm)				50			
Relative humidity middle layer (20–50 cm)				60			
Relative humidity deep layer (below 50 cm)				60			
Bedrock layer (below 200 cm)				Soil Wet Low			
**d) Settings of models’ spatial domain, resolution and orientation [urban case study areas]**							
Urban district	Model area—Grid			Size of the grid cells [m]			Model rotation (0° = from North … 180° = from South…)
	x	y	z	dx	dy	dz	
Compact low-rise [Casco Viejo]	150	128	26	0.75	0.75	2.0	24 °
Compact mid-rise [Abando/Indautxu]	165	120	26	1.45	1.45	2.0	17 °
Open-set high-rise [Txurdinaga/Miribilla]	165	120	34	3.0	3.0	2.0	9 °

**Table 7:** Materials setting applied in the *ENVI-met* model.

Surface	Buildings		Street/Path		Soil	
	Walls	Roofs	Pedestrian Path	Vehicular Path	Under Building	Under Grass
**Description**	Brick	Tile	Red brick stones	Asphalt road	Concrete (used/dirty)	Loamy soil
**Thickness [m]**	0.15	0.10	2	2	2	2
**U-value (W/m^2^K)**	0.44	0.84	NA	NA	NA	NA
**Albedo**	0.20	0.30	0.30	0.12	0.40	0.00

**Table 8:** Vegetation setting applied in the *ENVI-met* model.

Vegetation Element	Grass	Trees		
**Installation**	Street	Compact low-rise	Compact mid-rise	Open set high-rise
**Trees’ type and density**	Average dense	Platanus with 2/3 of full-fill crown’s density		
**Height [m]**	0.1	4	6	10
**Width [m]**	30% of the street’s width	1.5	4.5	6
**Albedo**	0.30	0.6	0.6	0.6

**Table 9:** Settings of the scenarios to study the effect of orientation (S0) and street’s pavement (S1).

Urban Area	Urban Canyon (H/W)	Scenario S0 Orientation in S0		Scenario S1	
		Street Pavement	Orientations	Street Pavement	Orientations
Compact low-rise	16 m /4.5 m (3.5)	Red brick stone	24° N-S	Red brick stone	N-S, NE-SW, SE-NW, E-W
Compact mid-rise	24 m /16 m (1.5)	Asphalt	17° N-S		
Open-set high-rise	40 m /33 m (1.3)	Asphalt	9° N-S		

**Table 10:** Settings of the scenarios M01 and M02 to study the effect of the vegetation elements.

Urban Area	Street Pavement	Orientations	Grass on the Street	Trees	Scenario M01		Scenario M02	
					Ht/H	Wt/W	Ht/H	Wt/W
Compact low-rise	Red brick stone	N-S, NE-SW, SE-NW, E-W	0.10 m	Tree 4 m; 1/2 without leaves	0.25	0.30	0.25	0.30
Compact mid-rise				Tree 6 m; 1/2 without leaves	0.25	0.28	0.25	0.30
Open-set high-rise				Tree 10 m; 1/2 without leaves	0.25	0.18	0.25	0.30

**Table 11:** Comparison of the PET peaks’ values, Sw, W_s_, the duration and the intensity of PET peaks and the duration of thermal discomfort between the orientation with the highest (NE–SW) and the lowest (NW–SE) PET values (data corresponds to the receptor R04 with pavement in red brick stones).

		Compact Low-Rise		Compact Mid-Rise		Open-Set High-Rise	
Orientation		NW–SE	NE–SW	NW–SE	NE–SW	NW–SE	NE–SW
**Intensity of PET peaks**	**°C**	31.90	52.97	27.59	47.26	24.34	45.80
**Short-wave direct irradiation**	**W/m^2^**	748.6	746.7	752.9	752.4	753.0	752.9
**Wind speed**	**m/s**	3.36	0.03	5.84	0.27	6.78	0.27
**Duration of the intensity of peaks period**	**hh.mm**	1.00 h (from 12:20 to 13:20)	1.00 h (from 15:30 to 16:30)	2.20 h (from 11:50 to 14:10)	2.20 h (from 14:40 to 17:00)	2.50 h (from 14:40 to 14:30)	2.40 h (from 14:30 to 17:10)
**Duration of thermal discomfort (PET > 23 °C)**	**hh.mm**	1.00 h (from 12:20 to 13:20)	11.00 h (from 09:10 to 20:30)	2.20 h (from 11:50 to 14:10)	10.30 h (from 09:50 to 20:20)	2.50 h (from 14:40 to 14:30)	10.20 h (from 10:00 to 20:20)

**Table 12:** Peak values of PET, T_s_, T_mrt_, T_a_, RH and W_s_ for all orientations with asphalt and red brick stones in compact mid-rise and open set high-rise urban canyons.

Urban Area	Material of the Pavement	Orientation	PET [°C]	T_mrt_ [°C]	T_s_ [°C]	T_a_ [°C]	RH [%]	Sw Dir [W/m^2^]	W_S_ [m/s]
**Compact mid-rise**	**Asphalt**	**N-S**	31.7	59.6	33.5	24.6	76.3	751.2	4.7
		**NE-SW**	48.0	66.6	44.1	23.9	70.3	738.5	0.4
		**NW-SE**	27.7	60.3	30.2	24.0	76.0	742.2	6.8
		**E-W**	32.6	62.0	30.5	24.8	76.6	589.1 a.m. 577.5 p.m.	4.9
	**Brick red stones**	**N-S**	33.5	64.6	31.2	24.4	76.3	751.2	4.7
		**NE-SW**	50.9	70.1	39.8	23.6	70.3	738.5	0.4
		**NW-SE**	29.2	65.4	28.6	24.0	76.0	742.2	6.8
		**E-W**	34.4	66.7	29.1	24.7	76.6	589.1 a.m. 577.5 p.m.	4.9
**Open-set high-rise**	**Asphalt**	**N-S**	29.2	61.0	36.5	22.9	68.7	750.2	5.0
		**NE-SW**	46.0	64.7	40.1	24.2	67.5	737.4	0.3
		**NW-SE**	25.4	60.4	33.7	22.7	67.4	742.0	6.8
		**E-W**	30.0	62.5	33.1	23.0	68.6	599.3 a.m. 592.9 p.m.	4.9
	**Brick red stones**	**N-S**	30.8	65.6	33.4	22.9	68.7	750.2	5.0
		**NE-SW**	48.3	69.2	38.5	23.8	67.5	737.4	0.3
		**NW-SE**	27.0	65.2	31.2	22.7	67.4	742.0	6.8
		**E-W**	31.6	67.0	30.8	23.0	68.6	599.3 a.m. 592.9 p.m.	4.9

**Table 13:** Peak values of PET, Mean Radiant Temperature (T_mrt_), Surface Temperature (T_S_), Air temperature (T_a_), Relative Humidity (RH) and Wind Speed (W_S_). Data extracted by all 14 receptors.

Urban Area	Scenario	Orientation	PET [°C]	T_mrt_ [°C]	T_S_ [°C]	T_a_ [°C]	RH [%]	W_S_ [m/s]
Compact low-rise	M01	N-S	33.4	61.5	26.4	23.3	74.0	2.6
		NE-SW	47.2	60.8	28.5	23.9	74.5	0.1
		NW-SE	31.6	62.2	25.5	23.1	72.5	3.0
		E-W	34.0	62.5	24.7	23.5	74.8	2.3
	M02	N-S	32.5	59.8	25.3	23.4	74.5	2.6
		NE-SW	45.8	59.0	26.9	23.9	74.3	0.1
		NW-SE	30.9	60.0	24.7	23.1	72.4	3.0
		E-W	30.3	56.8	23.4	23.6	73.7	2.3
Compact mid-rise	M01	N-S	32.8	62.9	26.9	24.3	77.3	3.5
		NE-SW	44.8	61.8	29..0	23.4	70.5	0.4
		NW-SE	27.4	62.5	26.5	23.9	76.6	4.6
		E-W	30.6	63.2	23.9	24.6	77.6	3.6
	M02	N-S	32.1	62.9	26.3	24.3	77.3	3.2
		NE-SW	43.5	59.6	29.0	23.4	70.5	0.4
		NW-SE	26.4	62.6	25.4	23.9	76.6	4.2
		E-W	28.8	59.9	23.2	24.7	77.6	3.2
Open set high-rise	M01	N-S	29.8	56.9	25.8	23.2	71.3	3.8
		NE-SW	40.6	57.1	29.2	23.5	67.7	0.3
		NW-SE	27.0	56.6	26.7	23.0	70.0	5.7
		E-W	29.6	56.3	23.8	23.3	71.3	3.8
	M02	N-S	28.9	56.6	25.8	23.1	71.0	3.2
		NE-SW	40.0	56.6	29.3	23.4	68.0	0.3
		NW-SE	25.5	57.0	26.3	22.8	68.4	4.6
		E-W	28.8	57.0	23.3	23.2	70.9	3.6

**Table 14:** PET peak values, for R01, R04 and R07 in compact lo-rise urban areas for the scenario with red brick stones, M01 (pavement in red brick stone and constant ratio Ht/H = 0.25) and M02 (pavement in brick red stone and constant ratio Ht/H = 0.25 and Wt/W = 0.30).

Urban Areas	Orient.	Rec.	[°C]	Thermal Perception	[°C]	∆ PET	Thermal Perception	[°C]	∆ PET	Thermal Perception
			S1		M01	S1—M01		M02	S1—M02	
Compact low-rise	N-S	R01	35.3	hot	32.6	2.7	warm	28.6	6.8	slightly warm
		R04	34.1	warm	26.4	7.7	slightly warm	24.1	10.1	slightly warm
		R07	34.7	warm	28.6	6.1	slightly warm	25.6	9.2	slightly warm
	NE-SW	R01	51.4	very hot	44.5	7.0	very hot	40.7	10.7	hot
		R04	53	very hot	41.7	11.3	Very hot	37.7	15.3	hot
		R07	52.8	very hot	43.5	9.2	very hot	40.3	12.4	hot
	NW-SE	R01	32.9	warm	29.9	3.0	warm	25.7	7.2	slightly warm
		R04	31.9	warm	27.3	4.6	slightly warm	22.7	9.2	comfortable
		R07	32.4	warm	27.0	5.4	slightly warm	24.3	8.2	slightly warm
	W-E	R01	35.0	hot	34.0	1.0	warm	30.2	4.8	warn
		R04	33.3	warm	27.2	6.1	slightly warm	25.3	8.0	slightly warm
		R07	34.5	slightly warm	31.0	3.6	warm	30.3	4.2	slightly warm

**Table 15:** Peak values of PET, for R01 (at the beginning of the urban canyon), R04 (in the middle) and R07 (at the end) in compact mid-rise urban areas for scenario S1, mitigation scenario M01 and M02.

Scenario	Orient.	Rec.	[°C]	Thermal Perception	[°C]	∆ PET	Thermal Perception	[°C]	∆ PET	Thermal Perception
			S1		M01	S1—M01		M02	S1—M02	
Compact mid-rise	N-S	R01	30.8	warm	26.5	4.2	slightly warm	25.1	5.7	slightly warm
		R04	31.7	warm	27.5	4.2	slightly warm	25.9	5.7	slightly warm
		R07	32.0	warm	27.2	4.8	slightly warm	25.7	6.3	slightly warm
	NE-SW	R01	50.5	very hot	40.8	9.8	hot	39.7	10.8	hot
		R04	49.5	very hot	39.0	10.5	hot	37.0	12.5	hot
		R07	47.5	very hot	37.4	10.0	hot	36.5	11.0	hot
	NW-SE	R01	28.3	warm	23.6	4.7	slightly warm	21.1	7.2	comfortable
		R04	29.1	warm	23.9	5.2	slightly warm	21.4	7.6	comfortable
		R07	27.8	warm	23.3	4.6	slightly warm	20.8	7.1	comfortable
	W-E	R01	32.6	warm	29.2	3.4	warm	28.4	4.2	slightly warm
		R04	31.2	warm	28.3	2.9	slightly warm	28.3	2.8	slightly warm
		R07	30.3	warm	27.3	3.1	slightly warm	27.4	3.0	slightly warm

**Table 16:** PET Peak values for R01 (at the beginning of the urban canyon), R04 (in the middle) and R07 (at the end) in the open-set high-rise urban areas for the scenario S1, M01 and M02.

Scenario	Orient.	Rec.	[°C]	Thermal Perception	[°C]	∆ PET	Thermal Perception	[°C]	∆ PET	Thermal Perception
			S1		M01	S1—M01		M02	S1—M02	
Open-set high-rise	N-S	R01	29.7	warm	24.7	5.0	slightly warm	25.0	4.7	slightly warm
		R04	29.2	warm	23.4	5.9	slightly warm	23.0	6.0	comfortable
		R07	30.8	warm	25.2	5.7	slightly warm	25.7	5.1	slightly warm
	NE-SW	R01	48.0	very hot	36.7	11.3	hot	33.8	14.1	warn
		R04	48.2	very hot	35.8	12.4	hot	32.8	15.4	warm
		R07	47.9	very hot	36.3	11.6	hot	33.4	14.6	warm
	NW-SE	R01	26.4	slightly warm	23.4	3.0	slightly warm	22.4	4.0	comfortable
		R04	25.8	slightly warm	22.6	3.2	comfortable	21.2	4.6	comfortable
		R07	27.0	slightly warm	23.7	3.3	slightly warm	22.8	4.2	comfortable
	W-E	R01	31.2	slightly warm	26.7	4.5	slightly warm	25.3	5.9	slightly warm
		R04	29.5	slightly warm	24.7	4.8	slightly warm	23.0	6.4	comfortable
		R07	29.8	slightly warm	25.9	3.9	slightly warm	24.4	5.4	slightly warm
